# Self-Reported Cancer Prevalence among Hispanics in the US: Results from the Hispanic Community Health Study/Study of Latinos

**DOI:** 10.1371/journal.pone.0146268

**Published:** 2016-01-25

**Authors:** Frank J. Penedo, Betina Yanez, Sheila F. Castañeda, Linda Gallo, Katy Wortman, Natalia Gouskova, Melissa Simon, William Arguelles, Maria Llabre, Lisa Sanchez-Johnsen, Carrie Brintz, Patricia Gonzalez, Linda Van Horn, Alfred W. Rademaker, Amelie G. Ramirez

**Affiliations:** 1 Department of Medical Social Sciences, Feinberg School of Medicine, Northwestern University, Chicago, IL, United States of America; 2 Institute For Behavioral and Community Health, Graduate School of Public Health, San Diego State University, San Diego, CA, United States of America; 3 Department of Psychology, San Diego State University, San Diego, CA, United States of America; 4 Department of Biostatistics, Gillings School of Public Health, University of North Carolina at Chapel Hill, Chapel Hill, NC, United States of America; 5 Department of Obstetrics & Gynecology, Feinberg School of Medicine, Northwestern University, Chicago, IL, United States of America; 6 Department of Psychology, University of Miami, Coral Gables, FL, United States of America; 7 Department of Psychiatry, University of Illinois at Chicago, Chicago, IL, United States of America; 8 Department of Preventive Medicine, Feinberg School of Medicine, Northwestern University, Chicago, IL, United States of America; 9 Institute for Health Promotion, University of Texas Health Science Center, San Antonio, TX, United States of America; State University of Maringá/Universidade Estadual de Maringá, BRAZIL

## Abstract

Cancer has surpassed heart disease as the leading cause of death among Hispanics in the U.S., yet data on cancer prevalence and risk factors in Hispanics in regard to ancestry remain scarce. This study sought to describe (a) the prevalence of cancer among Hispanics from four major U.S. metropolitan areas, (b) cancer prevalence across Hispanic ancestry, and (c) identify correlates of self-reported cancer prevalence. Participants were 16,415 individuals from the Hispanic Community Health Study/Study of Latinos (HCHS/SOL), who self-identified as Cuban, Dominican, Mexican, Puerto Rican, Central or South American. All data were collected at a single time point during the HCHS/SOL baseline clinic visit. The overall self-reported prevalence rate of cancer for the population was 4%. The rates varied by Hispanic ancestry group, with individuals of Cuban and Puerto Rican ancestry reporting the highest cancer prevalence. For the entire population, older age (OR = 1.47, *p* < .001, 95% CI, 1.26–1.71) and having health insurance (OR = 1.93, *p* < .001, 95% CI, 1.42–2.62) were all significantly associated with greater prevalence, whereas male sex was associated with lower prevalence (OR = 0.56, *p* < .01, 95% CI, .40-.79). Associations between study covariates and cancer prevalence also varied by Hispanic ancestry. Findings underscore the importance of sociodemographic factors and health insurance in relation to cancer prevalence for Hispanics and highlight variations in cancer prevalence across Hispanic ancestry groups. Characterizing differences in cancer prevalence rates and their correlates is critical to the development and implementation of effective prevention strategies across distinct Hispanic ancestry groups.

## Introduction

Hispanics/Latinos (hereafter referred to as Hispanics) are the largest and fastest growing ethnic group in the United States (U.S.). Numbering over 50 million, Hispanics comprised 16.3% of the U.S. population in 2010 and are estimated to account for 35% by 2050[1]. In 2009, cancer surpassed heart disease as the leading cause of death among Hispanics living in the U.S.[2] and accounted for 21% of all U.S. Hispanic deaths.

Relative to non-Hispanic Whites (NHWs), the Hispanic population shows substantial disparities in cancer prevalence, care, and outcomes[3]. Compared to NHWs, Hispanics in the U.S. have lower incidence rates for major cancers such as breast, lung, and prostate cancer; however, they have higher incidence rates for cervical and gastrointestinal cancers, which are typically associated with infections (e.g., Human Papillomavirus, hepatitis B) and are more prevalent in lower socioeconomic status (SES)[4] groups. Additionally, compared to NHWs, Hispanics are more likely to be diagnosed at an advanced stage of disease for most common cancers and have higher mortality rates for select cancers such as gastrointestinal, uterine, and cervical cancers,[5] largely due to lower rates of access to care, proper screening, and lack of early detection.

When examining cancer differences between NHWs and Hispanics, it is important to recognize that substantial variation may also exist within the Hispanic population as a function of Hispanic ancestry[4]. However, the available cancer incidence data for Hispanics in the U.S. are limited and poorly characterized in regard to country of origin, and generally focus on major ancestry groups (e.g., Mexicans, Puerto Ricans), thus overlooking other growing segments of the U.S. Hispanic population (e.g., Dominicans, Central and South Americans)[4]. Studies that have examined cancer incidence by Hispanic ancestry generally indicate that Cubans are comparable to NHWs in regard to incidence of the most common cancers[6]. Overall, prior work suggests that Mexicans have the lowest cancer incidence among all Hispanic ancestry groups. In contrast, Puerto Ricans consistently show the highest incidence rates of all Hispanic groups, particularly in regard to cervical, stomach and liver cancers[6]. Although sparse and limited in comprehensive assessment, previously published information regarding cancer incidence across Hispanic ancestry groups suggests that there may be important variations that warrant further investigation.

Differences in cancer incidence patterns among U.S. Hispanics may in part be due to substantial variation with respect to well-established factors associated with a cancer diagnosis (e.g., smoking, poor diet quality, physical inactivity, poor access to preventive care). Socioeconomic status, acculturation, migration back to country of origin for cancer care and other health behavior patterns[2,7–11] may also affect cancer incidence estimates. For example, limited access to health care and financial constraints observed in U.S. Hispanics have been associated with lower cancer screening rates[12,13,14]. Poor access to health care also limits opportunities for providers to counsel individuals on risks of tobacco use, poor nutrition, and physical inactivity[12]. The degree to which individuals have acculturated to the U.S. may also influence behavioral patterns related to a cancer diagnosis[11]. Acculturation, often assessed by English language acquisition, is related to the ability to understand physician recommendations and navigate the healthcare system in order to engage in preventive healthcare services[15,16]. However, because many studies that have characterized cancer rates among Hispanics have relied on existing cancer registries, information regarding acculturation, access to health care, and other important factors associated with cancer (e.g., diet) is limited.

In light of the lack of data on cancer rates among Hispanics, specifically Hispanic ancestry groups, the aim of this paper was to describe the prevalence of self-reported cancer diagnoses among Hispanics living in four U.S. metropolitan areas who participated in the Hispanic Community Health Study/Study of Latinos baseline examination. This study also characterizes how the prevalence of cancer diagnoses varies by Hispanic ancestry group and describes the relative associations of SES, age, sex, acculturation, health insurance, smoking, waist circumference, diet quality, self-reported physical activity, and cancer family history with cancer diagnoses in a cross-sectional sample.

## Materials and Methods

### Hispanic Community Health Study/Study of Latinos

The Hispanic Community Health Study/Study of Latinos (HCHS/SOL) is a prospective, population based study of the prevalence of multiple health conditions and their risk factors among 16,415 diverse Hispanics ages 18–74 residing in four U.S. metropolitan areas[17]. The four communities included in the HCHS/SOL are located in the Bronx, NY; Chicago, IL; Miami, FL; and San Diego, CA. Participants in the HCHS/SOL self-identified as Cuban, Dominican, Puerto Rican, Mexican, Central or South American. A stratified two-stage area probability sample of household addresses was selected in each of the four field centers. Stratification was based on concentration of Hispanic households, and low versus high SES (as measured by the proportion of persons with at least a high school education), each based on the 2000 decennial Census. Participants aged 45–74 years were over-sampled. Once a household was selected, all eligible household members were invited to participate. Persons who met the eligibility criteria and agreed to participate were scheduled for an IRB-approved consenting and assessment appointment at each field center clinic. The study followed all ethical standards for human research and was approved by the Institutional Review Board of each of the four field centers as follows: Albert Einstein College of Medicine Institutional Review Board (New York); Biomedical IRB of Northwestern University (Chicago); San Diego State University Institutional Review Board (San Diego); and the Social and Behavioral Sciences IRB at the University of Miami (Miami). Data presented in the form of frequencies are unweighted. Data presented in the form of percentage and means are weighted. Weighted data account for the disproportionate selection of the sample and to adjust for any bias due to differential nonresponse in the selected sample at the household and person levels. The weights were trimmed to limit precision losses due to their variability, and calibrated to the 2010 Census characteristics by age, sex and Hispanic ancestry within each field site’s target population. All analyses also account for cluster sampling and the use of stratification in sample selection. Complete details regarding the study design and procedures have been previously reported[17].

### Study Measures

Participants’ SES, age, sex, acculturation, health insurance status, smoking, diet, physical activity, family history of cancer and waist circumference measured by a trained member of the study staff were assessed. In order to measure SES, participants were asked to select one out of 10 possible income categories. Acculturation was assessed by the Language Use and Ethnic Social Relations subscales of the Short Acculturation Scale for Hispanics (SASH)[18]. For both SASH subscales, higher scores indicated greater acculturation and both subscales showed adequate reliability estimates (Cronbach’s α’s = .93 for Language Use and .72 for Ethnic Social Relations). Health insurance status included an item about current health insurance coverage (yes/no). Smoking was measured by history of smoking (current/former/never). Never smokers were defined as less than 100 lifetime cigarettes[19]. Waist circumference, modeled continuously, was used to account for abdominal obesity[20]. Diet quality was calculated as the 2010 Alternative Healthy Eating Index (AHEI), a composite score of diet quality based on foods and nutrients predictive of chronic disease risk[21]. The AHEI score was calculated from data obtained in 24-hour dietary recalls. Higher scores represent better diet. Physical activity was assessed using the Global Physical Activity Questionnaire (GPAQ) to quantify the number of minutes the participant engaged in moderate or vigorous activity related to work, transportation and/or leisure activities. This information was used to estimate a total metabolic equivalent task (METs) for each participant[22]. Higher scores represent more physical activity.

Familial history of cancer was assessed by asking the participant whether first-degree relatives such as a mother, father or sibling had ever been diagnosed with cancer. For analytic purposes, family cancer history was used as a binary indicator of a cancer diagnosis in a participant’s immediate family (mother, father, or sibling). Participant cancer diagnoses were assessed by self-report, where participants were asked whether “a doctor ever said that you have cancer or a malignant tumor?” Fourteen types of cancer (e.g., breast, prostate, colon, cervical, brain, etc.) and an “other” category were queried; participants responded “yes” or “no” to each type of cancer. This self-report method has shown a good degree of accuracy in prior cohort studies[23]. Age, acculturation, waist circumference, physical activity, diet, and income were modeled as continuous variables; all other variables were binary, with the exception of the three-category smoking status variable.

### Analyses

Descriptive statistics were used to characterize the sample. The prevalence of cancer diagnoses was calculated as a weighted proportion of participants who reported having been diagnosed with cancer at some point in their life and was then age-standardized to the 2010 U.S. Census. Prevalence of specific cancers was not age-standardized due to low sample sizes. For prevalence of cancer types (cervical, breast, colon, etc) by ancestry, percentage is reported as the percent of all cancers among that particular Hispanic ancestry group. A Rao-Scott chi-square test was used to detect a statistical difference in cancer prevalence among Hispanic ancestry groups. Follow-up between-groups chi-squared tests were used to compare statistical differences between each Hispanic ancestry group. Logistic regression was used to assess factors potentially associated with self-reported cancer diagnoses. Logistic regression was conducted using SAS 9.3 software (SAS PROC SURVEYLOGISTICS; SAS Institute, Cary, NC). All regression models accounted for the complex survey study design and used sampling weights. Missing data in covariates were handled by using multiple imputations. Eighteen percent of cases had missing data for one or more predictor variable. In order to address these missing data, twenty imputations were run using SAS PROC MI and fully conditional specification (FCS). Each missing value was modeled using all predictor variables in the main analysis model as well as the survey sample variables (primary sample unit, weight, and strata). Results from all models using multiple imputed data were summarized using SAS PROC MIANALYZE. Therefore, the only cases excluded from analysis were from participants missing information on a cancer diagnosis and those who endorsed multiple or “other” Hispanic backgrounds which altogether accounted for less than 4% of the sample.

Predictor variables that were entered into the logistic regression models were SES, age, sex, acculturation, health insurance status, smoking, waist circumference, diet, physical activity, and family history of cancer. All ten predictor variables were treated as covariates in the logistic regression models. The outcome was cancer prevalence.

A series of logistic regressions were used to investigate factors associated with cancer prevalence. An initial logistic regression model only included the covariates and cancer prevalence to determine relevant associations with cancer prevalence for the entire Hispanic population. A second logistic regression model included the covariates in addition to interactions of covariates with Hispanic ancestry group on cancer diagnoses. Findings from this model revealed significant interactions between Hispanic ancestry group and age, smoking, physical activity, and diet quality on cancer prevalence. A third model included covariates and only those interactions significant at the level of *p* ≤ .05 from model two. Given that recruitment site is confounded with Hispanic ancestry group (e.g., Cubans are predominantly recruited in Miami), recruitment site was added to a fourth logistic regression model to determine whether site was a significant correlate of cancer prevalence, and whether parameter estimates for Hispanic ancestry differed by greater than 10% between the third and fourth regression models. Because parameter estimates differed by more than 10% across models, we conducted a chi-square analysis to examine whether differences in cancer prevalence within Hispanic ancestry existed across the recruitment sites. Our findings revealed that there were no significant differences in cancer prevalence (*p* ≤ .05) within Hispanic ancestry compared across recruitment sites where there was a sufficient sample size for comparison. Therefore, all subsequent analyses do not include recruitment site as a model covariate. Only results from the first and third model are presented as the second model was considered an intermediate step necessary for the development of the third model. When interpreting findings from the final logistic regression analyses, a more stringent *p* ≤ 0.01 rather than *p* ≤ 0.05 was used to address family-wise error.

## Results and Discussion

### Participant Characteristics

From the overall sample of 16,415, a total of 15,802 (unweighted) fit the criteria for inclusion in analysis. Of the total 613 (unweighted) participants that were excluded, 523 were excluded because they identified with more than one or “other” Hispanic ancestry (e.g., South American and Cuban), 37 were excluded because their response to the question about having had a cancer diagnosis was missing, and 53 were excluded because they identified with more than one or “other” Hispanic ancestry and had missing data on the cancer diagnosis question. Table 1 contains descriptive statistics for all the study variables and cancer prevalence rates for the target population. The mean age of the target population was approximately 40 years. The population consists of slightly more females than males. The majority of the population reported an annual household income of less than $30,000. Half of the population reported having health insurance. Current and former smokers account for less than half of the population and a minority of the population reported that an immediate family member (mother, father, or sibling) had been diagnosed with cancer. Scores on the SASH subscales indicated that on average the population was low in acculturation regarding language use and ethnic social relations.

**Table 1:** Descriptive statistics for study variables and cancer prevalence^1^.

	Central American		Cuban		Dominican		Mexican		Puerto Rican		South American		Total	
	n = 1,725		n = 2,344		n = 1,475		n = 6,461		n = 2,721		n = 1,076		n = 15,802	
	n	%	n	%	n	%	N	%	N	%	n	%	n	%
18–39 yrs. old	612	53%	504	33%	473	52%	2193	56%	689	42%	311	44%	**4782**	**47%**
40–59 yrs. old	893	36%	1312	43%	766	37%	3323	35%	1416	42%	584	43%	**8294**	**38%**
60 or older	220	10%	528	24%	236	11%	945	10%	616	17%	181	13%	**2726**	**14%**
Female	1044	53%	1247	48%	963	60%	4019	53%	1584	50%	639	55%	**9496**	**52%**
Income > $30k	394	27%	518	27%	353	28%	2222	40%	840	35%	337	35%	**4664**	**34%**
Overall reported lifetime cancer prevalence (any)	57	3.15%	129	4.86%	39	3.24%	220	3.13%	147	5.22%	37	3.12%	**629**	**4.06%**
Breast	9	21%	15	12%	14	36%	42	13%	30	16%	10	23%	**120**	**16%**
Prostate	7	16%	11	11%	6	14%	11	5%	11	6%	3	10%	**49**	**8%**
Lung	1	4%	1	0%	2	9%	3	3%	6	11%	1	2%	**14**	**4%**
Colorectal	2	3%	9	7%	1	1%	7	2%	8	4%	1	4%	**28**	**4%**
Blood/lymph glands	2	4%	2	1%	1	2%	15	11%	7	4%	2	4%	**29**	**5%**
Uterine	9	10%	14	9%	3	4%	22	6%	17	19%	7	11%	**72**	**10%**
Melanoma	0	0%	9	7%	0	0%	6	1%	1	1%	0	0%	**16**	**3%**
Liver	0	0%	1	0%	1	1%	5	2%	4	1%	0	0%	**11**	**1%**
Brain	1	2%	1	0%	0	0%	4	2%	5	6%	0	0%	**11**	**2%**
Stomach	2	3%	1	0%	0	0%	9	4%	5	5%	1	1%	**18**	**3%**
Cervical	15	25%	26	15%	6	23%	70	30%	28	18%	6	12%	**151**	**21%**
Testicular	0	0%	0	0%	0	0%	6	13%	0	0%	0	0%	**6**	**4%**
Skin (non- melanoma)	2	2%	22	17%	1	1%	8	4%	8	10%	3	16%	**44**	**10%**
Bone	0	0%	0	0%	0	0%	1	0%	3	2%	0	0%	**4**	**0%**
Other	11	25%	30	28%	5	12%	44	16%	40	21%	8	35%	**138**	**22%**
Mother Cancer Diagnosis	181	9%	305	11%	130	8%	711	10%	384	13%	134	12%	**1845**	**11%**
Father Cancer Diagnosis	121	6%	340	13%	102	6%	487	6%	298	9%	93	7%	**1441**	**8%**
Brother or Sister Cancer Diagnosis	118	5%	229	8%	89	4%	485	6%	268	7%	89	6%	**1278**	**6%**
Mother, Father, or Sibling Cancer Diagnosis ^2^	371	18%	722	27%	274	16%	1445	19%	781	25%	269	22%	**2862**	**22%**
Never Smoked Cigarettes	1191	71%	1169	54%	1112	78%	4140	64%	1292	49%	705	65%	**9609**	**61%**
Former Cigarette User	297	15%	488	19%	207	11%	1346	18%	562	17%	227	22%	**3127**	**17%**
Current Cigarette User	234	15%	683	27%	155	11%	963	18%	860	34%	142	13%	**3037**	**21%**
Health Insurance (Yes)	547	31%	909	42%	1043	71%	2848	42%	2138	78%	393	42%	**7878**	**50%**
	**M**	**95% CI**	**M**	**95% CI**	**M**	**95% CI**	**M**	**95% CI**	**M**	**95% CI**	**M**	**95% CI**	**M**	**95% CI**
Age	39.77	0.96	46.59	1.05	39.13	1.32	38.57	0.73	42.90	0.98	42.46	1.49	41.33	0.49
Waist circumference (cm)	95.08	0.79	97.49	0.83	95.65	1.49	97.66	0.63	99.74	1.03	93.44	1.17	97.35	0.42
SASH Ethnic Social Relations^3^	2.08	0.05	1.99	0.03	2.30	0.04	2.22	0.03	2.51	0.03	2.24	0.05	2.22	0.02
SASH Language	1.72	0.09	1.59	0.06	2.00	0.10	2.07	0.05	3.12	0.08	1.79	0.07	2.10	0.05
Diet Quality^4^	47.11	0.40	43.92	0.29	48.41	0.52	51.98	0.35	41.57	0.35	45.99	0.61	47.50	0.34
Physical Activity	3.83	0.36	2.39	0.22	3.38	0.51	3.79	0.25	3.62	0.37	3.12	0.41	3.40	0.15

Note

^1^Overall cancer prevalence is based on self-report and age-standardized to the 2010 US Census. Self-reported prevalence of specific cancers is not age-standardized due to low sample size. For cancer types (cervical, breast, colon, etc) percentage is reported as the percent of all cancers among that particular Hispanic ancestry group.

^2^Some participants had both a mother and a father with a cancer diagnosis but were only counted once in the immediate family variable.

^3^SASH is the Short Acculturation Scale for Hispanics, range is 1–5.

^4^Range is 0–110.

### Cancer Prevalence

Overall, four percent of the population reported having received a cancer diagnosis at some time in their lives. For the total population, the most common cancers were “other” (i.e., other cancers not specifically queried such as head and neck, hematological and renal carcinoma), followed by cervical and breast cancer (see Table 1). The Rao-Scott chi- square test indicated a significant difference among Hispanic ancestry groups with respect to overall cancer prevalence (*x*^2^ = 41.18, *p* < .001). Follow-up chi-square analyses revealed that Cubans and Puerto Ricans had significantly higher overall reported cancer prevalence rates compared to all other Hispanic ancestry groups (*p*s < .01); however, Cubans and Puerto Ricans did not significantly differ from one another.

### Correlates of Cancer Prevalence

Table 2 contains the odds ratios from the first model which is the logistic regression model containing the covariates and cancer diagnoses for all Hispanics. Results indicate a statistically significant effect for age, sex, and health insurance on cancer prevalence. For all Hispanics, each 10-year increase in age was associated with higher odds of having been diagnosed with cancer. Compared to females, Hispanic males had lower odds of having been diagnosed with cancer. Hispanics with health insurance had higher odds of having been diagnosed with cancer. Follow-up descriptive statistics revealed that among Hispanics diagnosed with cancer, 28.6% did not have health insurance whereas 71.4% were insured. Among Hispanics not diagnosed with cancer, 50.5% did not have health insurance and 49.5% were insured. Although the finding was marginally significant at the *p* < .01 level (*p* = .0102), Hispanics with an immediate family member with cancer had higher odds of having been diagnosed with cancer.

**Table 2:** Logistic Regression Odds Ratios for Covariates on Cancer Prevalence for Model containing all Hispanics (N = 15,802).

Parameter	p	Odds Ratio	Lower CI	Upper CI
Model Intercept	**< .001**	0.004	0.001	0.01
Age (per 10 year increase)	**< .001**	1.47	1.26	1.71
Sex (Male)	**<0.01**	0.56	0.40	0.79
Income > $30k	0.16	0.95	0.89	1.02
Former Cigarette User	0.07	1.38	0.97	1.97
Current Cigarette User	0.64	0.90	0.57	1.41
Physical Activity	0.31	0.98	0.95	1.01
Health Insurance (Yes)	**< .001**	1.93	1.42	2.62
Family Diagnosis of Cancer	0.02	1.32	1.04	1.67
SASH Social ^1^	0.35	0.87	0.66	1.16
SASH Language^1^	0.20	1.13	0.94	1.37
Waist Circumference	0.61	1.01	0.97	1.06
Diet Quality	0.83	1.01	0.98	1.03

Note.^.^

^1^ SASH is the Short Acculturation Scale for Hispanics.

Findings from the third model contain study covariates and only those Hispanic ancestry by risk interactions significant at the level of *p* < .05 from model two. Results indicated a finding for diet quality. Although the odds ratio for the main effect of diet quality was not significant at the *p* < .01 level (OR = 1.02; .99–1.05 95% CI), the association of diet quality with cancer prevalence varied significantly by Hispanic ancestry such that for each one-point increase on the AHEI 2010 diet quality measure, Dominicans had lower odds (OR = .86; .79-.93 95% CI) of having been diagnosed with cancer relative to Mexicans.

This study sought to report the cancer prevalence rate in a diverse sample of Hispanics living in four U.S. metropolitan areas participating in the Hispanic Community Health Study/Study of Latinos (HCHS/SOL), the largest epidemiologic study of U.S. Hispanics’ health to date. An additional goal of this study was to determine the relative associations of age, sex, SES, acculturation, health insurance status, smoking, waist circumference, diet quality, physical activity, and family history of cancer with lifetime cancer prevalence. Furthermore, the study evaluated differences in factors associated with cancer prevalence across six Hispanic ancestry groups. While prior studies have reported the prevalence or incidence of cancer in the U.S. Hispanic population, most of these studies have been based on cancer registries with limited ethnic self-identification and have lacked a comprehensive set of SES, acculturation, and behavioral risk factor (e.g., smoking, diet, etc.) indicators[4,5]. Our findings indicate that four percent (n = 629) of the population reported a diagnosis of cancer at any point in their lives. The cancer prevalence in the current study is greater than the 2.27% age-adjusted 19-year point prevalence reported in by the Surveillance, Epidemiology, and End Results Program (SEER)[24]. The difference in these numbers may be accounted by the fact that the SEER data reflects a 19-year time frame whereas our study examined prevalence of cancer at any point in the participants’ lives. Nonetheless, although our data were weighted to the mean age of Hispanics at each recruitment site, which is a younger average age than the U.S. population, our cancer prevalence rates remained higher relative to SEER[24].

Our cross-sectional findings regarding cancer self-reported prevalence are consistent with previous longitudinal research indicating that Cubans and Puerto Ricans have the highest incidence relative to the other four Hispanic ancestry groups[6,25]. Findings regarding associations with cancer prevalence for the entire Hispanic population indicated that older age, female sex, and having health insurance were all associated with a diagnosis of cancer. The significant association we found between older age and cancer diagnosis is consistent with previous findings[2,12,26,27]. However, that women are more likely to be diagnosed with cancer than men is inconsistent with SEER data showing that Hispanic men are more likely to be diagnosed with cancer[2]. This finding could be explained by the high number of cervical cancer diagnoses reported by participants, which is consistent with previous research that Hispanic women are more likely to be diagnosed with cervical cancer than non-Hispanic White women[2]. Another possible explanation for the greater number of females diagnosed with cancer is that female participants were younger than male participants, and as females are diagnosed with cancer at an earlier age than males[28], the broad age range in our sample likely contributed to this difference. This broad age range in our sample may have also contributed to the lower prevalence of breast cancer relative to cervical cancer, as women with cervical cancer are more likely to be diagnosed at a younger age than women with breast cancer[29]. Our findings also showed an association between health insurance status and cancer prevalence, which is most likely an indirect relationship explained by cancer screening and access to health care. Although cancer screening behavior data were not available for this study, one possibility is that individuals with health insurance have greater cancer screening rates and access to physician visits that increase the likelihood of cancer detection, whereas individuals without health insurance have fewer opportunities to be screened and receive a cancer diagnosis. It is also possible that individuals with a history of cancer elected to enroll in health insurance as a result of their diagnosis. Our findings also indicate that a better quality diet is associated with lower odds of a cancer diagnosis for Dominicans when compared to Mexicans. Future longitudinal studies focused on Hispanic group differences in diet quality may shed some light into diet quality and cancer risk.

The present study contributes to our current understanding of cancer prevalence in Hispanics in the U.S. in several ways. First, our findings indicate that sociodemographic factors such as sex and insurance status are factors associated with cancer prevalence for Hispanics. Other sociodemographic factors such as income and socio-cultural factors such as acculturation were not associated with cancer prevalence for Hispanics. Second, our findings show that there are differences in the cancer prevalence rate by Hispanic ancestry groups, even after adjusting for covariates. Finally, although diet quality was not significantly associated with cancer prevalence for the entire Hispanic sample, results show that diet quality may be differentially related to a cancer diagnosis for certain Hispanic ancestry groups but not others.

Despite the contributions of our findings, there are several limitations that must be noted. Eighteen percent of the sample had missing data on at least one of the covariates, which is a limitation of the data. However, these missing data were addressed with multiple imputations which allowed us to maximize use of our data. Given the cross-sectional nature of the study, our results should be interpreted with caution. Our models are not causal and although conceptually guided by well-established literature on factors associated with cancer, it is very likely that having been diagnosed with cancer affected several behaviors associated with cancer. Therefore, future studies that aim to describe the predictive utility of factors associated with cancer among Hispanic ancestry groups should involve longitudinal designs that allow for evaluation of prospective associations or predictive models. Furthermore, the 4% cancer prevalence rate and limited number of cancer diagnoses across each cancer site did not allow us to explore differences in prevalence and factors associated with cancer across unique cancer sites. It is also important to note that this study reported cancer prevalence as opposed to incidence and as a result these data do not capture Hispanics who were diagnosed with cancer but did not survive through the point of study entry. As such, prevalence rates might reflect potential differences in cancer diagnoses among Hispanics or reflect differences in the survival patterns of cancers due to participants diagnosed with more aggressive cancer such as lung cancer (more common among men) expiring before participation in this study or the latency between behaviors such as smoking and the development of cancer. Given the long latency period between smoking and cancer, coupled with the relative young age of the sample, participants may not have developed cancer by the time of study participation. Therefore, more longitudinal research is needed to better investigate determinants of the development of cancer over time among Hispanics. Finally, cancer prevalence was determined by self-report. Due to high rates of uninsured, low SES participants, it is likely that cancer screening was less than optimal and therefore possible that a significant number of our participants had undetected cancers that we were not able to capture.

## Conclusions

Hispanics in the U.S. encompass a growing heterogeneous population with varying cancer prevalence and risk attributed to access to health care status and age. Although behavioral factors such as smoking are linked to cancer [2,30], findings from our study did not reveal significant relationships with smoking and cancer prevalence. The lack of significant findings regarding smoking may be due to the cross-sectional nature of our study, underscoring the need for longitudinal research focusing on diverse Hispanics living in the U.S. To develop and implement effective prevention strategies, it is critical that we understand how risk factors and their contribution to cancer prevalence may vary across distinct Hispanic ancestry groups. It is clear that access to health care continues to be a major factor associated with cancer prevalence among Hispanics in the U.S. As the Affordable Care Act is implemented, a growing number of Hispanics will be accessing the U.S. health care system and undergoing screening for the most common cancers. Future evaluation of cancer prevalence across Hispanic ancestry within the context of universal health care access will provide insight into whether disparities across Hispanic groups persist despite such access.
